# Synaptic Diversity Suppresses Complex Collective Behavior in Networks of Theta Neurons

**DOI:** 10.3389/fncom.2020.00044

**Published:** 2020-05-26

**Authors:** Lucas Lin, Ernest Barreto, Paul So

**Affiliations:** ^1^Department of Computer Science, Stanford University, Stanford, CA, United States; ^2^Department of Physics and Astronomy and Interdisciplinary Program in Neuroscience, George Mason University, Fairfax, VA, United States

**Keywords:** network, synaptic diversity, theta neuron, oscillations, synchronization, heterogeneity

## Abstract

Comprehending how the brain functions requires an understanding of the dynamics of neuronal assemblies. Previous work used a mean-field reduction method to determine the collective dynamics of a large heterogeneous network of uniformly and globally coupled theta neurons, which are a canonical formulation of Type I neurons. However, in modeling neuronal networks, it is unreasonable to assume that the coupling strength between every pair of neurons is identical. The goal in the present work is to analytically examine the collective macroscopic behavior of a network of theta neurons that is more realistic in that it includes heterogeneity in the coupling strength as well as in neuronal excitability. We consider the occurrence of dynamical structures that give rise to complicated dynamics via bifurcations of macroscopic collective quantities, concentrating on two biophysically relevant cases: (1) predominantly excitable neurons with mostly excitatory connections, and (2) predominantly spiking neurons with inhibitory connections. We find that increasing the synaptic diversity moves these dynamical structures to distant extremes of parameter space, leaving simple collective equilibrium states in the physiologically relevant region. We also study the node vs. focus nature of stable macroscopic equilibrium solutions and discuss our results in the context of recent literature.

## 1. Introduction

In 1949, Hebb (1949) proposed that cell assemblies are the true functional unit of the nervous system. The cerebral cortex contains networks of neuronal assemblies that comprise a large number of interacting neurons (Harris, 2005; Sporns et al., 2005). Individual neuronal assemblies organize via transient synchronization to generate collective behavior that is critical to communication between the neuronal assemblies themselves. Furthermore, it has been suggested that this synchronous neural activity, as well as average spatiotemporal firing patterns that emerge from these populations, are important coding mechanisms (Harris, 2005).

In developing an analytical understanding of the behavior of large neuronal assemblies, it is prohibitively challenging to use realistic models of actual neurons. To make progress, it is useful to use a canonical model that can represent the general behavior of a whole class of neurons (Izhikevich, 2000). A model can be considered canonical for a family of models if a continuous change of variables can transform any instance of that family into the canonical model. Such a model is advantageous due to its universality since any behavior exhibited by the canonical model informs the behavior of the entire family of neurons. In approaching the characterization of neuronal populations specifically, the use of a canonical model is beneficial in that it may be amenable to a complete analytical treatment.

Physiologically, excitable neurons are typically classified into two types (Hodgkin, 1948; Izhikevich, 2007). Here, we are concerned with Type I neurons, which represent a category that includes cortical excitatory pyramidal neurons that generate action potentials at an arbitrarily low rate when a sufficiently large input stimulus is applied. Ermentrout and Kopell derived a one-dimensional neuronal model that includes a saddle-node bifurcation on an invariant circle, or SNIC bifurcation, and demonstrated that it is canonical for Type I neurons near the firing threshold (Ermentrout and Kopell, 1986). We use this model, also termed the theta neuron, to analyze the collective dynamics of a large population of Type I neurons.

Instead of being concerned with the exact values of all neuronal state variables in a large network of model neurons, we look to classify the macroscopic or collective behaviors that describe the activity of a population as a whole. Much early work studied such collective behaviors in terms of mean firing rates. Famously, the Wilson–Cowan equations consider a homogeneous population of interconnected excitatory and inhibitory neurons (Wilson and Cowan, 1972, 1973; Coombes, 2006). But, in recent years, many authors have employed the groundbreaking techniques of Ott and Antonsen (2008, 2009), which yield an understanding of the collective dynamics from the asymptotic behavior of a low-dimensional set of reduced equations for an appropriate set of macroscopic variables.

Luke et al. (2013) used these methods to analyze a network of globally-coupled theta neurons (see also Luke et al., 2014; So et al., 2014). These authors analytically obtained the asymptotic dynamics of a Kuramoto-type order parameter that quantifies the collective network dynamics. This work was later adapted to a spatiotemporal context by Laing (2014, 2015) and used to make a connection between the microscopic theta neuron steady states and the corresponding mean-field firing-rate-based model. At about the same time, similar work was pursued independently by Pazó and Montbrió (2014) for pulse-coupled Winfree networks. Then, Montbrió et al. (2015) used similar analytic techniques to describe the collective dynamics of a population of quadratic integrate-and-fire (QIF) neurons in terms of the network firing rate and average membrane potential. It is important to note that the theta neuron can be transformed into a QIF neuron by an appropriate change of variable. Montbrió et al. (2015) went further, linking networks of these neurons by identifying a conformal mapping between the two macroscopic variables for the QIF network (i.e., firing rate and mean membrane potential) and the Kuramoto order parameter for the theta neuron system.

The current work builds directly on the results of Luke et al. (2013), which included heterogeneity in the excitability parameter of the theta neuron in order to model this obviously significant feature of real neuronal ensembles. However, the neurons were assumed to be linked together with a single value of coupling strength. In the current work, we extend this analysis to also include synaptic diversity, modeled as heterogeneity in the coupling strength parameter. Our aim is to determine how this additional and realistic feature of the network model affects the macroscopic patterns produced by the population as a whole. We note that in Appendix E1 of Montbrió et al. (2015), this situation was also considered for QIF networks, and we comment on the relationship of our work to theirs in section 4.

We also take interest in the nature of equilibrium solutions of the macroscopic network variables. Luke et al. (2013) noted that collective stable node and stable focus solutions exist, and that their nature can be identified by observing the collective network response to a perturbation (see their Figure 5), since the relaxation back to a focus solution involves oscillatory behavior in the macroscopic variable. Recently, di Volo and Torcini (2018) (see also Bi et al., 2020) argued that collective oscillations in balanced spiking inhibitory networks can arise via this mechanism when driven by appropriate fluctuations. They showed using a model based on Montbrió et al. (2015) that the frequency of such collective oscillations match the relaxation dynamics around a stable focus equilibrium. Thus we are also interested in examining how introducing synaptic diversity affects the node vs. focus nature of macroscopic equilibrium solutions.

## 2. Methods

### 2.1. Microscopic Formulation

The theta neuron model is a canonical representation of a Type-1 neuron (Ermentrout, 2008) and is given by

$$\overset{∙}{\theta }=\left(1-cos\theta \right)+\eta \left(1+cos\theta \right),$$

where the phase angle θ characterizes the state of the neuron. The neuron is considered to “spike,” or produce an action potential, when θ crosses π while increasing. We call η the “excitability parameter” and think of it as playing the role of a fixed input current. If η < 0, then the model has a stable and an unstable equilibrium which we call the resting state and the threshold, respectively. In this situation, the neuron is excitable, as a sufficiently large external stimulus could push the phase of the neuron across the unstable equilibrium, where upon θ would travel around the circle, pass θ = π and spike, and then approach the stable equilibrium from the other side. As η increases, the stable and unstable equilibria get closer together, merge in a SNIC bifurcation at η = 0, and disappear. For η>0, the neuron's dynamics is that of a limit cycle, representing a periodically spiking neuron.

We consider a network of *N* theta neurons,

(1) $$\begin{array}{r} \overset{∙}{\theta_{j}}=\left(1-cos\theta_{j}\right)+\left[\eta_{j}+I_{syn,j}\right]\left(1+cos\theta_{j}\right), \end{array}$$

where *j* = 1, …, *N* is the index of the *j*-th neuron. The theta neurons are coupled together via a pulse-like synaptic current *I*_*syn, j*_ given by

(2) $$\begin{array}{r} I_{syn,j}=\frac{k_{j}}{N}\overset{N}{\underset{i=1}{\sum }}P_{n}\left(\theta_{i}\right), \end{array}$$

where $P_{n}\left(\theta \right)=a_{n}{\left(1-cos\theta \right)}^{n},n\in \mathbb{N}$, *k*_*j*_ is the coupling strength, and *a*_*n*_ is a normalization constant such that

$$\overset{2\pi }{\underset{0}{\int }}P_{n}\left(\theta \right)d\theta =2\pi .$$

In this model, the parameters η_*j*_, *k*_*j*_, and *n* represent biological features. η_*j*_ determines either the degree to which neuron *j* is excitable (for η_*j*_ < 0), or the frequency of regular spiking (for η_*j*_>0). *k*_*j*_ describes the strength of coupling between neuron *j* and its presynaptic partners, and can be inhibitory (*k*_*j*_ < 0) or excitatory (*k*_*j*_>0). The parameter *n* determines the shape of the synaptic current. As *n* increases, the current pulse becomes more sharply peaked. Throughout most of this paper, we set *n* = 2, but we also consider *n* = 9 as noted.

To quantify the macroscopic collective behavior of the network, we use the usual Kuramoto complex order parameter *z*(*t*):

$$z\left(t\right)=\frac{1}{N}\overset{N}{\underset{j=1}{\sum }}e^{i\theta_{j}}.$$

This is the centroid of the phase distribution. Perfect phase synchrony corresponds to |*z*| = 1, and partial phase synchrony to 0 < |*z*| <1. Note, however, that because the angular speed of a spiking theta neuron is not uniform in θ, a population of such neurons exhibits a degree of phase synchrony with |*z*|≠0 when completely uncoupled.

Since neurons in real biological networks exhibit a range of intrinsic excitabilities, the parameter η_*j*_ is typically different for each neuron. New in this work, we also allow for diversity in the coupling strengths *k*_*j*_. We model this by drawing these parameters at random from two independent Cauchy–Lorentz distribution functions *g*_η_(η) and *g*_*k*_(*k*) given by

(3) $$\begin{array}{r} g_{\eta }\left(\eta \right)=\frac{1}{\pi }\frac{\Delta_{\eta }}{{\left(\eta -\eta_{0}\right)}^{2}+\Delta_{\eta }^{2}},g_{k}\left(k\right)=\frac{1}{\pi }\frac{\Delta_{k}}{{\left(k-k_{0}\right)}^{2}+\Delta_{k}^{2}}, \end{array}$$

where η_0_ and *k*_0_ are the centers of the distributions, and Δ_η_ and Δ_*k*_ are their half-widths at half-maximum. The latter two parameters describe the degree of heterogeneity in the excitability parameter and the coupling strength, respectively. This particular choice of distribution function permits analytical solutions. Because the distribution has infinite support, the infinitely large networks include both positive and negative η's and *k*'s, meaning that the network contains a mixture of excitable and spiking neurons as well as inhibitory and excitatory connections. The ratios of these depend on the values of η_0_ and *k*_0_, i.e., where the distributions are centered.

### 2.2. Mean Field Reduction

We adopt a mean-field continuum description of our network (Kuramoto, 1975, 1984) by considering the limit *N* → ∞ such that the network is described by a probability density function *F*(θ, η, *k, t*), where *F*(θ, η, *k, t*)*dθdηdk* gives the probability at time *t* of finding an oscillator with phase in [θ, θ+*dθ*] and parameters in [η, η+*dη*] and [*k, k*+*dk*]. The total number of neurons is conserved and we assume that the marginal probability distribution functions *g*_η_(η) and *g*_*k*_(*k*) are both time-independent and independent of each other. Thus, *F* satisfies the continuity equation,

(4) $$\begin{array}{r} \frac{\partial F}{\partial t}+\frac{\partial }{\partial \theta }\left(Fv_{\theta }\right)=0, \end{array}$$

where *v*_θ_ represents the velocity of a neuron and is given by the continuum version of the single neuron equation,

(5) $$\begin{array}{l} v_{\theta }=(1-cos\theta )+[\eta +ka_{n}\int_{-\infty }^{\infty }\int_{-\infty }^{\infty }\int_{0}^{2\pi }F(\theta^{\prime },\eta^{\prime },k^{\prime },t)\\ \;{\left(1-cos\theta^{\prime }\right)}^{n}d\eta^{\prime }dk^{\prime }d\theta^{\prime }](1+cos\theta ). \end{array}$$

We also define the order parameter *z*(*t*) in the continuum limit,

(6) $$\begin{array}{r} z\left(t\right)\equiv \overset{2\pi }{\underset{0}{\int }}d\theta \overset{\infty }{\underset{-\infty }{\int }}d\eta \overset{\infty }{\underset{-\infty }{\int }}dkF\left(\theta ,\eta ,k,t\right)e^{i\theta }. \end{array}$$

This describes the collective behavior of the infinite network.

Ott and Antonsen showed that in the continuum limit, the macroscopic behavior of Kuramoto-type populations of globally coupled and heterogeneous phase oscillators displays low-dimensional dynamics (Ott and Antonsen, 2008, 2009). They adopted the ansatz that the probability density function describing the network can be written as a Fourier expansion in the phase variable whose coefficients are powers of a single complex function. Using the continuity equation and a self-consistency argument, they derived an equation that this complex function must satisfy. Ultimately, with appropriate choices of *g*_η_ and *g*_*k*_ (such as Equation 3), this procedure leads to a low-dimensional ordinary differential equation (ODE) whose asymptotic dynamics coincides with that of the order parameter *z*(*t*) of the infinite discrete network. Thus, the asymptotic collective dynamics of the infinite discrete network can be obtained by solving that low-dimensional ODE instead of the infinitely many coupled ODEs of the discrete network (i.e., Equation 1), or the partial differential equation that describes the network in the continuum description (i.e., Equation 4). Later, Marvel et al. (2009) showed that the Ott-Antonsen (OA) approach applies more generally to other oscillator-type systems for which the velocity field *v*_θ_ can be written in “sinusoidally coupled form,” i.e., $v_{\theta }=fe^{i\theta }+h+f^{*}e^{-i\theta }$, where the dependence on the individual oscillator's phase occurs only through the first harmonics *e*^*iθ*^ and *e*^−*iθ*^.

These methods were applied to a globally-coupled population of theta neurons with heterogeneity in the excitability parameter, which can be written in the sinusoidally-coupled form described above, by Luke et al. (2013). The result was a two-dimensional (i.e., complex) ordinary differential equation for *z*(*t*) which identifies the asymptotic collective dynamics of the infinite discrete network. The equation admits three possible asymptotic states: equilibrium solutions with either real (node) or complex-conjugate (focus) eigenvalues, and limit cycles. The authors confirmed by numerical simulation that the reduced model accurately captures the collective behavior of discrete networks of 10, 000 neurons.

Note that there is considerable discussion in the literature regarding the interesting question of the marginal stability of the OA manifold and its relation to earlier work. See, for example, Pikovsky and Rosenblum (2015), Mirollo (2012), Watanabe and Strogatz (1994), Watanabe and Strogatz (1993), and Goldobin and Dolmatova (2019), and for networks with parameter-dependent oscillators, such as our theta neuron network, see Pietras and Daffertshofer, 2016.

In the following, we follow the approach in Luke et al. (2013), but include heterogeneity in the coupling strength *k* as in Equation (3). We comment on the relationship between our results and those in Appendix E1 of Montbrió et al. (2015) in section 4.

### 2.3. Bifurcation Analysis Methods

In addition to constructing standard one-dimensional bifurcation diagrams, we employ the following less-common approach to bifurcation analysis (Luke et al., 2013). With *z*(*t*) = *x*(*t*)+*iy*(*t*) and fixed values of *n* and Δ_*k*_, we think of the conditions for an equilibrium solution (*x*_*e*_, *y*_*e*_),

(7) $$\begin{array}{rcl} ẋ\left(t\right)=f\left(n,\Delta_{k};x_{e},y_{e},\eta_{0},\Delta_{\eta },k_{0}\right) & = & 0\\ ẏ\left(t\right)=g\left(n,\Delta_{k};x_{e},y_{e},\eta_{0},\Delta_{\eta },k_{0}\right) & = & 0, \end{array}$$

as being two constraints on the five independent variables *x*_*e*_, *y*_*e*_, η_0_, Δ_η_, and *k*_0_. A saddle-node bifurcation occurs when one of the eigenvalues of the Jacobian *J* of the equations of motion (Equations 7) is zero. Thus, it is sufficient to require

(8) $$\begin{array}{r} det\left[J\left(x_{e},y_{e},\eta_{0},\Delta_{\eta },k_{0}\right)\right]=0. \end{array}$$

With this equation, we have three constraints on five variables, thus defining two-dimensional surfaces. We manipulate these equations to find expressions for η_0_, Δ_η_, and *k*_0_, each in terms of *x*_*e*_ and *y*_*e*_. This then allows us to parametrically plot the saddle-node bifurcation surfaces in the three-dimensional parameter space (η_0_, Δ_η_, *k*_0_) by scanning over (*x*_*e*_, *y*_*e*_). In other words, we construct plots of two-dimensional surfaces in the parameter space (η_0_, Δ_η_, *k*_0_) such that points on these surfaces correspond to parameter values at which an (unspecified) equilibrium undergoes a saddle-node bifurcation.

Since our reduced system is two-dimensional, surfaces of Hopf bifurcations can be obtained in the same way by replacing Equation (8) with

(9) $$\begin{array}{r} tr\left[J\left(x_{e},y_{e},\eta_{0},\Delta_{\eta },k_{0},\Delta_{k}\right)\right]=0 \end{array}$$

subject to

(10) $$\begin{array}{r} det\left[J\left(x_{e},y_{e},\eta_{0},\Delta_{\eta },k_{0},\Delta_{k}\right)\right]>0. \end{array}$$

Finally, surfaces corresponding to node-focus (NF) transitions can be obtained, for two-dimensional systems, using the condition

(11) $$\begin{array}{r} tr{\left[J\right]}^{2}-4det\left[J\right]=0. \end{array}$$

In the following, we examine how the saddle-node, Hopf, and node-focus transition surfaces evolve as Δ_*k*_ changes.

### 2.4. Computational Methods

One-dimensional bifurcation diagrams of the reduced equations were calculated using XPPAUT (Ermentrout, 2002), and three-dimensional diagrams were generated with custom-made code using the ParametricPlot3D function in Mathematica Version 12.0 (Wolfram Research, 2019). In addition, simulations of the discrete network were carried out to confirm the validity of our results, but are not reported here.

## 3. Results

### 3.1. The Reduced System

To derive the reduced dynamical system for our network, we follow the methods of Ott and Antonsen (2008, 2009), Marvel et al. (2009), and Luke et al. (2013), but include heterogeneity in the coupling strength according to Equation (3). We sketch the procedure here.

We first write the velocity equation in sinusoidally coupled form, $v_{\theta }=fe^{i\theta }+h+f^{*}e^{-i\theta }$, with

(12) $$\begin{array}{l} f=-\frac{1}{2}[(1-\eta )-kH(z,n)]\\ h=(1+\eta +kH(z,n)), \end{array}$$

where *H*(*z, n*) is the rescaled synaptic current (Luke et al., 2013)

$$\begin{array}{ccc} H\left(z,n\right) & =a_{n}\left(A_{0}+\overset{n}{\underset{q=1}{\sum }}A_{q}\left(z^{q}+z^{*q}\right)\right) & \\ A_{q} & =\overset{n}{\underset{j,m=0}{\sum }}\delta_{j-2m,q}Q_{jm}\\ Q_{jm} & =\frac{{\left(-1\right)}^{j-2m}n!}{2^{j}m!\left(n-j\right)!\left(j-m\right)!}. \end{array}$$

Next we adopt the OA ansatz that the solution to the continuity equation, *F*, can be written as a Fourier series,

(13) $$\begin{array}{r} F\left(\theta ,\eta ,k,t\right)=\frac{g\left(\eta ,k\right)}{2\pi }\left\{1+\overset{\infty }{\underset{q=1}{\sum }}\left(\alpha^{*}{\left(\eta ,k,t\right)}^{q}e^{iq\theta }+\alpha {\left(\eta ,k,t\right)}^{q}e^{-iq\theta }\right)\right\}, \end{array}$$

where *g*(η, *k*) = *g*_η_(η)*g_*k*_(*k*) is the joint probability distribution for the two independent random variables. At this point, the complex function α(η, *k, t*) is yet to be determined. This manifold is invariant if and only if |α(η, *k, t*)| <1 and α satisfies

(14) $$\begin{array}{r} \overset{∙}{\alpha }=i\left(f\alpha^{2}+h\alpha +f^{*}\right). \end{array}$$

Substituting Equation (13) into Equation (6) [which defines the order parameter *z*(*t*)] then gives

$$z\left(t\right)=\overset{\infty }{\underset{-\infty }{\int }}\overset{\infty }{\underset{-\infty }{\int }}\alpha \left(\eta ,k,t\right)g\left(\eta ,k\right)d\eta dk,$$

which can be evaluated using analytic continuation and the residue theorem, resulting in

(15) $$\begin{array}{r} z\left(t\right)=\alpha \left(\eta_{0}+i\Delta_{\eta },k_{0}+i\Delta_{k},t\right). \end{array}$$

Substituting Equations (**??**) for *f* and *h* into Equation (14), combining with Equation (15), and evaluating at the residue, the reduced dynamical system is obtained:

(16) $$\begin{array}{l} \dot{z}=-i\frac{{\left(z-1\right)}^{2}}{2}+\frac{{\left(z+1\right)}^{2}}{2}[-\left(\Delta_{\eta }+\Delta_{k}H(z,n)\right)\\ \;+i\left(\eta_{0}+k_{0}H(z,n)\right)]. \end{array}$$

This result is similar to the result in Luke et al. (2013), but the incorporation of heterogeneity in the parameter *k* adds the relatively simple extra term that involves Δ_*k*_. We numerically verified that predictions obtained with Equation (16) match the asymptotic collective behavior exhibited by a large discrete network of theta neurons. In fact, we find that the predictions from the reduced system are quite valid for networks with as few as 10, 000 neurons (see also Luke et al., 2013). Note that we only consider solutions to Equation (16) with |*z*| ≤ 1.

### 3.2. The Effects of Synaptic Diversity

As the title suggests, our main result is that increasing the synaptic diversity by increasing the parameter Δ_*k*_, which is the width of the coupling strength distribution given in Equation (3), reduces the complexity of the collective dynamics of the network. We illustrate this result by using Equation (16) to construct series of one-dimensional bifurcation diagrams with increasing Δ_*k*_. We then provide a more comprehensive perspective by using sequences of three-dimensional bifurcation diagrams.

Luke et al. (2013) argued that typically, interesting dynamics happen—by which we mean the occurrence of bifurcations of macroscopic quantities—when there is a competition between the intrinsic dynamics of individual neurons and the synaptic input. Thus, we concentrate attention on two generic cases. In our Case 1, we consider the situation in which most neurons are excitable (η_0_ < 0) and are coupled by mostly excitatory synapses (*k*_0_>0). Case 2 considers predominantly spiking neurons (η_0_>0) with mostly inhibitory coupling (*k*_0_ < 0). We keep *n* = 2 until the end, where we check the effects of setting *n* = 9.

#### 3.2.1. One-Dimensional Bifurcation Diagrams

**Figure 1:**
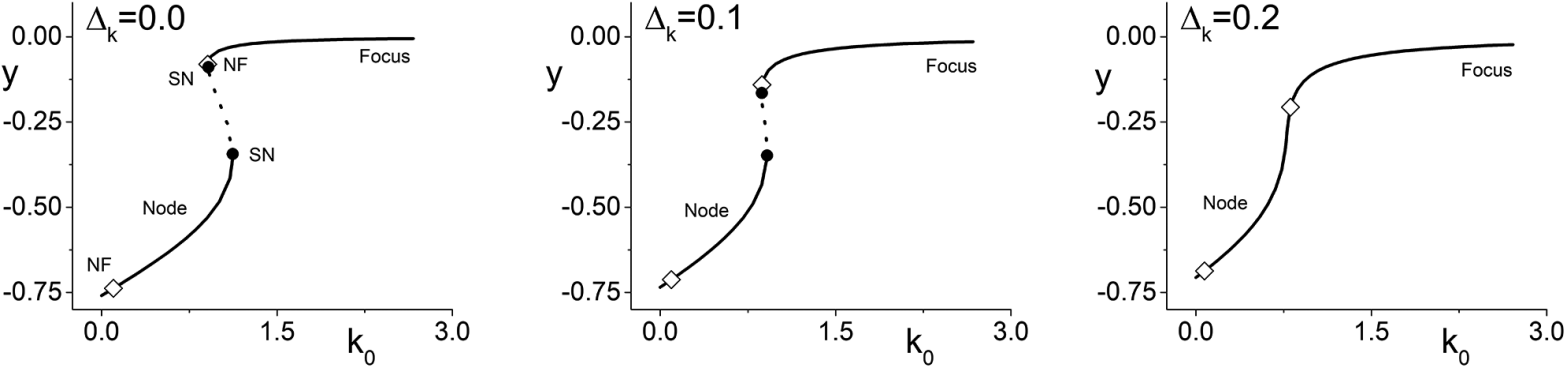
Case 1: One-dimensional bifurcation diagrams showing *y* = *Im*(*z*) vs. the parameter *k*_0_, which is the center of the coupling strength distribution. The panels show the diagrams for increasing values of Δ_*k*_, the width of the coupling strength distribution. Stable (unstable) equilibria are represented by solid (dotted) lines, and are nodes or foci as indicated. Open diamonds are node-focus transitions. For Δ_*k*_ = 0.0, two saddle-node bifurcations are seen (solid black circles). These merge and disappear as Δ_*k*_ increases, but the node-focus transitions remain. The other parameters are η_0_ = −0.3, Δ_η_ = 0.08, and *n* = 2.

We begin by considering Case 1 (excitable neurons with excitatory coupling). Figure 1 (left) shows a bifurcation diagram of *y* = *Im*(*z*) vs. the parameter *k*_0_ for Δ_*k*_ = 0, i.e., no diversity in the coupling strength between neurons. The solid lines represent stable equilibria. Equilibria on the lower branch are nodes, and most of the upper branch are foci. The dotted line indicates unstable equilibria, and the solid circles are saddle-node bifurcations. The stable node that emerges from the upper saddle-node bifurcation almost immediately transitions into a stable focus at the location marked with an open diamond (NF). (Observe also that there is another node-focus transition near *k*_0_ = 0.0.) Thus, throughout this range of *k*_0_, the collective dynamics of the network is attracted to an equilibrium state. Interestingly, however, there is an interval of *k*_0_ for which different equilibrium states coexist.

The middle and right panels of Figure 1 show the same diagram but for Δ_*k*_ = 0.1 and 0.2, respectively. We see the saddle-node points merge and disappear, thus removing the interval of multistability from these diagrams (with other parameters fixed). In this sense, introducing synaptic diversity removes an interesting dynamical feature from the network's behavior. Below we examine if this is true more globally. Note, however, that the node-focus transition points remain.

**Figure 2:**
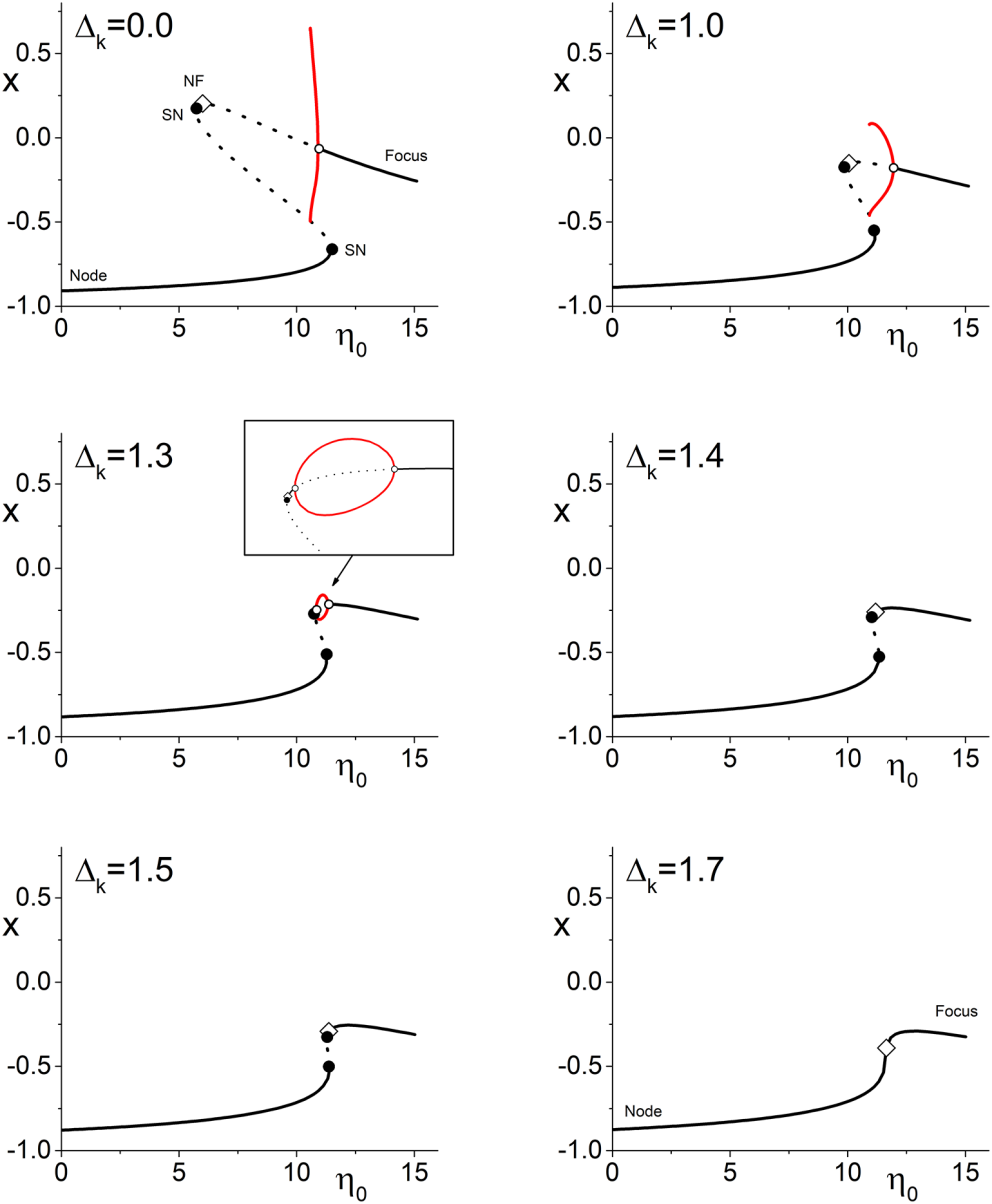
Case 2: One-dimensional bifurcation diagrams showing *x* = *Re*(*z*) vs. the parameter η_0_. The panels show the diagram for increasing values of Δ_*k*_, the width of the coupling strength distribution. Stable (unstable) equilibria are represented by solid (dotted) black lines, and are nodes or foci as indicated. Open diamonds are node-focus transitions. The maximal and minimal values of *x* on stable limit cycles are represented by the red lines. Solid black circles are saddle-node bifurcations, and open circles are Hopf bifurcations. As Δ_*k*_ increases, the various bifurcations merge and disappear, but the node-focus transition remains. The other parameters are *k*_0_ = −9.0, Δ_η_ = 0.5, and *n* = 2.

Figure 2 illustrates the more complicated situation that arises in Case 2 (spiking neurons with inhibitory coupling). Here, the upper left panel shows the one-dimensional bifurcation diagram of *x* = *Re*(*z*) vs. η_0_ for Δ_*k*_ = 0 (no coupling strength diversity). We see a structure of lines representing stable and unstable equilibria (nodes and foci as indicated) with saddle-node bifurcations and a node-focus transition, which is very similar to that in Figure 1. In addition, however, there is a supercritical Hopf bifurcation depicted by the open circle, along with the limit cycle that emerges from it as η_0_ decreases. This attracting limit cycle indicates that the network can exhibit collective time-dependent behavior with a degree of phase synchrony that oscillates in time. In the diagram, the red lines are the maximum and minimum values of *x* on this limit cycle. At its largest extent, the limit cycle collides with an unstable equilibrium in a homoclinic bifurcation. Note also that there is again an interval of multistability. In this case, the lower stable equilibrium (node) coexists with either the limit cycle or the upper equilibrium (focus), depending on η_0_.

The subsequent panels show the same diagram but for increasing values of the coupling strength diversity parameter Δ_*k*_ as indicated. Again, we see that the various bifurcation points approach each other as Δ_*k*_ increases. An interesting phenomenon is how the homoclinic point approaches the upper saddle-node bifurcation. By Δ_*k*_ = 1.3, it has disappeared, and a new Hopf bifurcation is seen on the upper branch (this sequence of events indicates that we are near a Bagdanov–Takens point, where the saddle-node, homoclinic, and left Hopf bifurcation coincide). The limit cycle now forms a loop linking the two Hopf points—see the magnified view in the inset.

As before, we observe that all these complexities merge and annihilate as Δ_*k*_ increases further. The two Hopf points coalesce, eliminating the limit cycle and the unstable equilibrium sandwiched between them. Subsequently the two remaining saddle-node points merge and disappear. Thus we see again that introducing synaptic diversity diminishes the dynamical repertoire of the network (at least when holding other parameters fixed). But, as before, the node-focus transition persists.

#### 3.2.2. Three-Dimensional Bifurcation Diagrams

A reasonable question is whether or not this “decomplexification” by increasing synaptic diversity is something that happens locally in a particular region of parameter space, or if it is a more global phenomenon. We address this by showing three-dimensional bifurcation diagrams that incorporate the structures shown in Figures 1 and 2.

**Figure 3:**
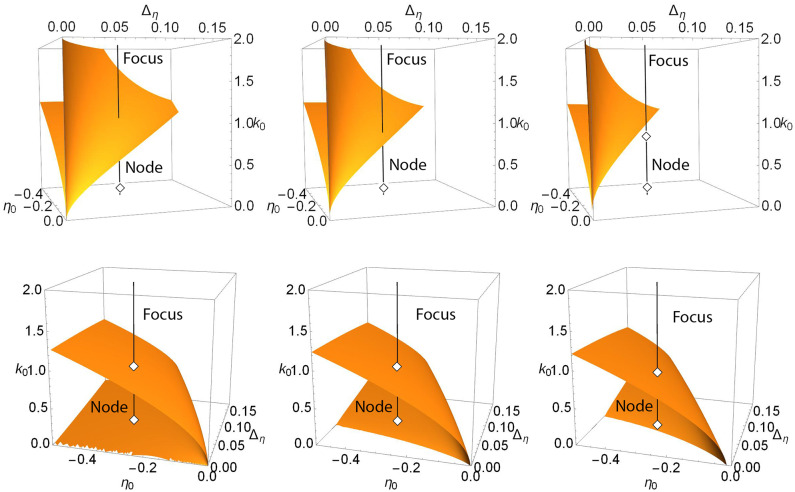
Case 1: Plots of the saddle-node surface (top) and the node-focus surface (bottom) for Δ_*k*_ = 0.0 (left), 0.1 (middle), and 0.2 (right), with *n* = 2. The black lines correspond to η_0_ = −0.3 and Δ_η_ = 0.08, and show the path traversed along *k*_0_ in the bifurcation diagrams shown in Figure 1. Equilibria along the lines are labeled node or focus; see the discussion in the text. In the upper sequence, a hidden node-focus transition (open diamond) emerges in the right panel.

For example, the upper panels in Figure 3 show a more general view of Case 1. The top left panel shows a locus of saddle-node points embedded in the (η_0_, Δ_η_, *k*_0_) parameter space for Δ_*k*_ = 0. This appears as a V-shaped folded sheet with a sharp crease. The black line corresponds to η_0_ = −0.3 and Δ_η_ = 0.08 and is the path traversed along *k*_0_ in the one-dimensional bifurcation diagram shown in Figure 1 (left). Node and focus equilibria along the black line are as indicated. This black line can be seen to intersect the saddle-node surfaces in two points; these are the same two saddle-node points shown in Figure 1 (left). The remaining upper panels of Figure 3 match those of Figure 1, and one can see that by increasing Δ_*k*_, the saddle-node surface moves to the left (i.e, toward more negative η_0_ and smaller Δ_η_). In so doing, the creased fold in the surface approaches the fixed black line and then moves beyond it, so that the intersection points merge and then disappear. In the right panel, there is no longer any intersection.

Recall that in Figure 1, a node-focus point occurs very close to the upper saddle-node bifurcation. In the perspectives shown in the upper left and middle panels of Figure 3, this point is not visible, but it emerges in the right panel for Δ_*k*_ = 0.2.

The lower panels in Figure 3 show the node-focus surface for the same situation, but rotated to better show the folded shape. The diamonds show where the black line intersects this surface, and are the same diamonds that mark the NF transitions on the black lines in the upper panels. Within the region of parameter space shown, one generally finds a single attracting focus above (higher *k*_0_) the NF surface, and an attracting node within the fold. However, multistability can occur.

**Figure 4:**
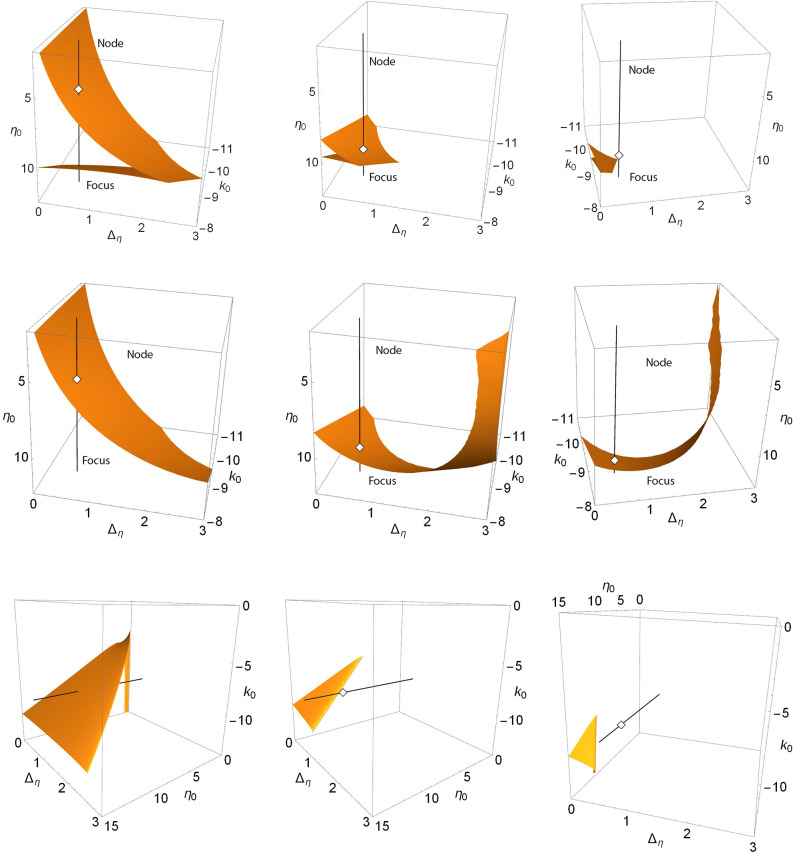
Case 2: Plots of the saddle-node surface (top), the node-focus surface (middle), and the Hopf surface (bottom) for Δ_*k*_ = 0.0 (left), 1.3 (middle), and 1.7 (right), with *n* = 2. The black line corresponds to *k*_0_ = −9.0 and Δ_η_ = 0.5, and is the path traversed along η_0_ in the bifurcation diagrams shown in Figure 2. Open diamonds are node-focus transitions. The views in the upper and middle right panels have been rotated for clarity. In particular, the black line does not intersect the SN surface for Δ_*k*_ = 1.7. The view in the lower panels is also rotated to better show the structure.

For Case 2, a similar sequence of events can be seen in the upper three panels of Figure 4. These show the saddle-node surfaces corresponding to the Δ_*k*_ = 0.0, 1.3, and 1.7 panels of Figure 2. Here, the black line is fixed at *k*_0_ = −0.9 and Δ_η_ = 0.5, representing the path traversed along η_0_ in the one-dimensional bifurcation diagrams of Figure 2. Again we see a folded and creased saddle-node surface that migrates toward the unphysical negative Δ_η_ region with increasing Δ_*k*_ until it no longer intersects the black line. Note that in the right panel, the view has been rotated to show the lack of intersection.

The middle panels show the node-focus surface. As Δ_*k*_ increases, the surface lowers and twists, but remains present. The larger structure, of which we only see limited portions here, is difficult to discern from these images. Below we examine a more comprehensive view.

The lower panels of Figure 4 show the corresponding Hopf surfaces. The view has been rotated to give an easier-to-understand perspective. In the left panel, this surface resembles a high-heeled shoe, and we see a single intersection with the black line. This intersection point is the same Hopf bifurcation denoted with the open circle in Figure 2 for Δ_*k*_ = 0. As Δ_*k*_ increases, the “shoe” migrates into the unphysical negative-Δ_η_ region, leaving the black line without intersections. Note that the NF transition point, hidden behind the surface in the left panel, emerges in the middle and right panels.

**Figure 5:**
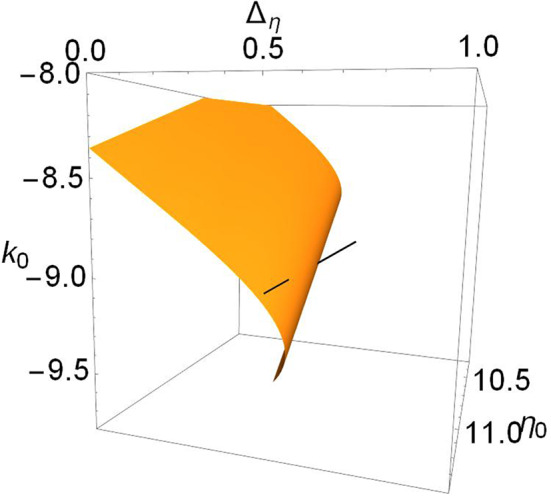
Magnified view of the Hopf surface shown in the lower middle panel of Figure 4, for Δ_*k*_ = 1.3, showing two intersections.

Recall that for Δ_*k*_ = 1.3, we saw the interesting structure with the two Hopf bifurcation points in Figure 2. This case corresponds to the lower middle panel of Figure 4. Since it is hard to see, we present in Figure 5 a magnification of the region where the black line intersects the surface. We see that the Hopf surface has a gentle curl at the edge such that as the surface migrates away, the approaching curl gives rise to a second intersection point before the surface goes away entirely. In fact, the lower edge of the Hopf surface seen here is a line of Bagdanov–Takens bifurcations.

**Figure 6:**
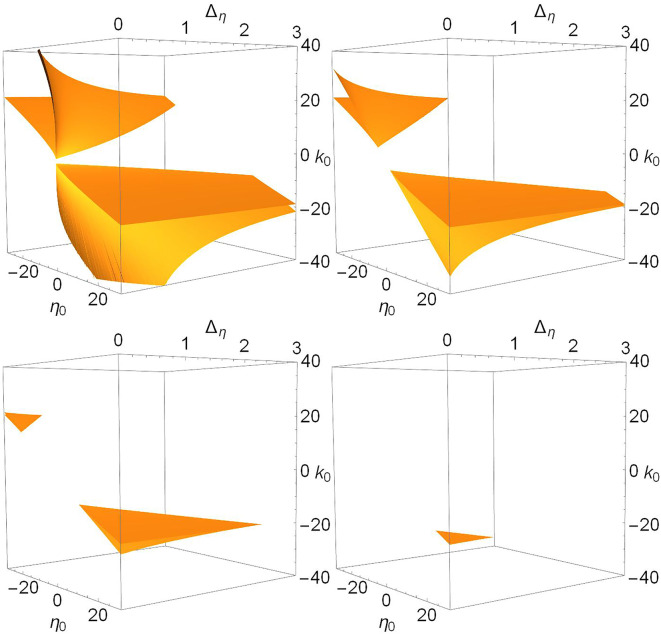
The saddle-node surfaces disappear from view as Δ_*k*_ increases. *n* = 2 and Δ_*k*_ = 0.0 **(top left)**, 1.0 **(top right)**, 2.0 **(lower left)**, and 3.0 **(lower right)**.

**Figure 7:**
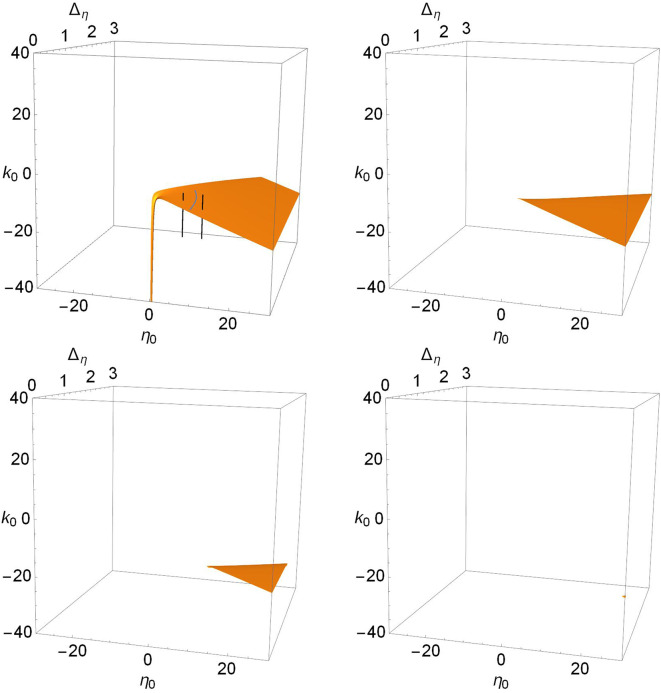
The Hopf surfaces disappear from view as Δ_*k*_ increases. *n* = 2 and Δ_*k*_ = 0.0 **(top left)**, 1.0 **(top right)**, 2.0 **(lower left)**, and 3.0 **(lower right)**. The gray line on the surface in the top left panel marks the boundary between sub- and super-critical Hopf bifurcations, and the black lines correspond to paths taken to create the one-dimensional bifurcation diagrams shown in Figure 8. Super-critical bifurcations occur on the side with larger η_0_, and in all the other panels.

To complete the story, we show in Figures 6, 7, and **9** views of the saddle-node, Hopf, and node-focus surfaces with the ranges of η_0_, *k*_0_, and Δ_*k*_ greatly expanded. For visual clarity, we did not expand the Δ_η_ range, but our conclusions still hold. Recall also that Δ_η_ is the half-width at half-maximum of the distribution *g*_η_ in Equation (3). Therefore, negative values of this parameter are not considered.

In Figure 6, we see that the saddle-node surfaces are actually two V-shaped sharply-creased sheets corresponding our two cases. One surface occurs in the negative-η_0_/positive-*k*_0_ region, matching Case 1 (excitable neurons coupled with excitation), and the other occurs in the positive-η_0_/negative-*k*_0_ region, matching Case 2 (spiking neurons coupled with inhibition). Note also that the edges of the creased folds bend away toward ±η_0_ as Δ_η_ increases. As Δ_*k*_ increases, the two folded sheets migrate away from each other until essentially nothing is left within the view shown.

In contrast, we see in Figure 7 that there is only one Hopf surface. It resides entirely within the Case 2 region (positive-η_0_/negative-*k*_0_). There is no corresponding Hopf surface in the Case 1 region (negative-η_0_/positive-*k*_0_).

**Figure 8:**
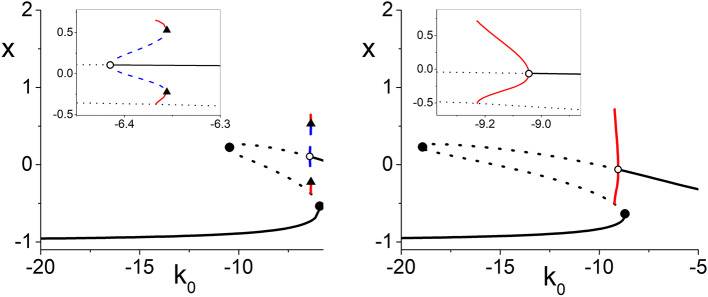
One-dimensional bifurcation diagrams showing *x* = *Re*(*z*) vs. *k*_0_ illustrating subcritical **(left)** and supercritical **(right)** Hopf bifurcations (open circles). The insets are magnifications. These diagrams correspond to paths along the black lines in the top left panel of Figure 7. Here, black solid (dotted) lines are stable (unstable) equilibria, red (dotted blue) lines indicate stable (unstable) limit cycles, solid circles are saddle-node bifurcations, and triangles are saddle-node-of-periodic-orbits bifurcations. The other parameters are Δ_*k*_ = 0.0, *n* = 2, Δ_η_ = 0.4, and η_0_ = 6.0 **(left)** and 11.0 **(right)**.

The gray curved line in the top-left panel of Figure 7 is the boundary between the subcritical and supercritical Hopf bifurcations. The supercritical versions occur on the side with larger values of η_0_. In all the other panels, only supercritical bifurcations are found. The black lines correspond to paths used to calculate the one-dimensional bifurcation diagrams shown in Figure 8. This latter figure shows the periodic orbits that emerge from the Hopf bifurcations. On the left we see a subcritical bifurcation, where the dotted blue line denotes an unstable periodic orbit. Note that as this unstable orbit grows with increasing *k*_0_, it collides with a stable periodic orbit (red line) at a saddle-node-of-periodic-orbits bifurcation (triangle). From this point, the stable orbit grows with decreasing *k*_0_ until it collides with the lower unstable equilibrium and disappears in a homoclinic bifurcation. The right panel shows a supercritical Hopf bifurcation, with a stable periodic orbit (red) emerging and growing with decreasing *k*_0_ until it too disappears in a homoclinic bifurcation.

Returning to the more comprehensive view of Figure 7, we see that as Δ_*k*_ increases, the surface disappears into the unphysical negative Δ_η_ region. Thus, increasing the synaptic diversity also removes the Hopf bifurcation structure such that by Δ_*k*_ = 3.0, essentially nothing is left within the view shown. Interestingly, we find that subcritical Hopf bifurcations only occur for small values of Δ_*k*_, i.e., little synaptic strength diversity. For example, the subcritical Hopf bifurcation shown in Figure 8 (left) for Δ_*k*_ = 0.0 remains subcritical as Δ_*k*_ increases to 0.114, where it merges with the saddle-node-of-periodic-orbits bifurcation at a Bautin point. Increasing Δ_*k*_ further, the bifurcation becomes supercritical, and goes on to follow a scenario similar to that shown in Figure 2, until it disappears at Δ_*k*_ = 0.864.

**Figure 9:**
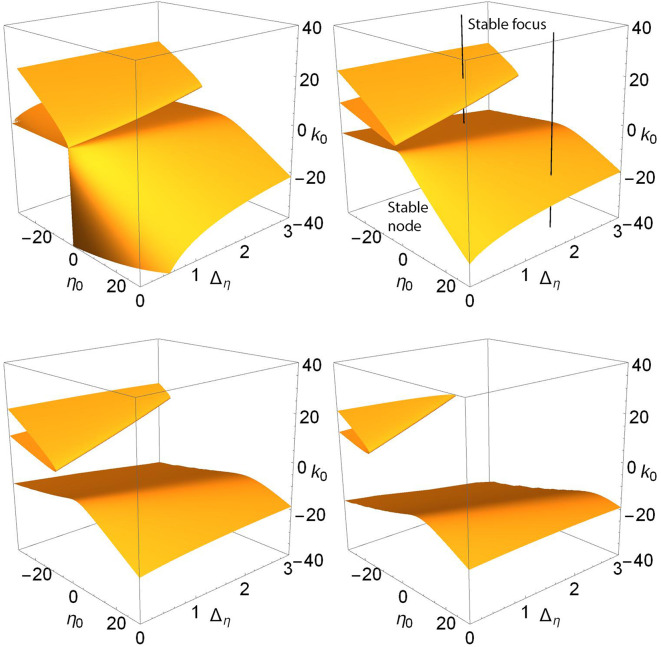
The node-focus surfaces as Δ_*k*_ increases. *n* = 2 and Δ_*k*_ = 0.0 **(top left)**, 1.0 **(top right)**, 2.0 **(lower left)**, and 3.0 **(lower right)**. Along the black lines in the upper right panels, we find a stable focus for *k*_0_ above the surfaces, and a stable node below; see the discussion in the text.

Finally, we show the node-focus transition surfaces as Δ_*k*_ increases in Figure 9. The structure looks complicated for Δ_*k*_ = 0.0, but its overall shape becomes clear as Δ_*k*_ increases and its various components separate. The surfaces occur in two pieces. There is a folded-over sheet in the Case 1 region of excitable neurons (η_0_ < 0) with excitation (*k*_0_>0), and another sheet that covers the entire η_0_-Δ_η_ region shown and which, for Δ_*k*_ = 0.0, dips sharply down toward negative *k*_0_ in the Case 2 region (η_0_>0 and *k*_0_ < 0; spiking neurons with inhibition). As Δ_*k*_ increases, two things happen: the folded sheet in the Case 1 region migrates away toward the negative-η_0_ direction, and the other sheet flattens out (i.e., occurs within a more restricted range of *k*_0_ within the view shown).

To understand the nature of the equilibrium solutions that correspond to this region of parameter space, we identified and followed the equilibria along the black lines seen in the upper right panel of Figure 9. Generally, for parameters corresponding to the region above (meaning higher values of *k*_0_) the surfaces shown, there exists a single stable focus equilibrium (recall that we restrict attention to solutions with |*z*| ≤ 1). For the line with positive η_0_, the NF surface is crossed only once as *k*_0_ decreases, and below it we find a stable node. For the line with negative η_0_, there are three surface crossings as *k*_0_ decreases. We observe the following sequence of equilibria: Stable focus, stable node (within the folded upper surface), stable focus (between the folded surface and the lower sheet), and stable node (below the lower sheet). The same scenarios were observed along lines shifted to Δ_η_ = 0.5, and for the different values of Δ_*k*_ of the other panels (not shown). Note, however, that in some cases saddle-node bifurcations create other coexisting equilibria—compare Figure 6—so it is not entirely clear from Figure 9 alone which equilibrium transitions as the NF surface is crossed. (One may resolve this issue with one-dimensional bifurcation diagrams such as those in Figures 1 and 2.) However, as we have seen, the other bifurcation surfaces leave this region of parameter space as Δ_*k*_ increases, and the situation becomes simpler.

It is interesting to compare our two cases in this context. In the Case 1 region, the folded sheet introduces more node-focus transitions. And as the synaptic diversity Δ_*k*_ increases, this folded surface moves away toward negative η_0_, thus leading to reduced complexity within the view shown. However, the lower sheet persists, and covers the entire η_0_-Δ_η_ plane. It shifts to be within a more restricted interval of *k*_0_ (i.e., it flattens), but it remains.

**Figure 10:**
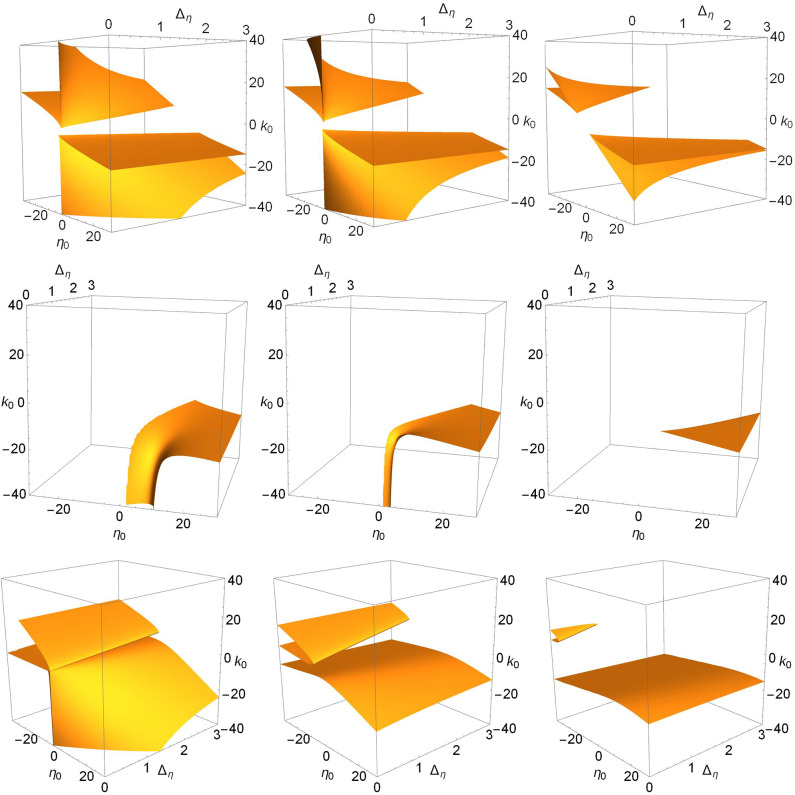
The surfaces for *n* = 9, for which the synaptic pulse (Equation 2) is much narrower. **(Top)** The saddle-node surfaces for Δ_*k*_ = 0.0, 1.0, 2.0. **(Middle)** The Hopf surfaces for Δ_*k*_ = 0.0, 1.0, 2.0. **(Bottom)** The node-focus surfaces for Δ_*k*_ = 0.0, 3.0, 5.0 (values chosen for visual clarity).

Finally, we consider the effect of changing *n* = 2 to *n* = 9 in Equation (2), which results in a much narrower synaptic pulse. Figure 10 shows (top to bottom) the saddle-node, Hopf, and node-focus surfaces for increasing values of Δ_*k*_ (right to left). In general, the surfaces are very similar to those shown in Figures 6, 7, and 9. The most obvious difference is in the Hopf surface for Δ_*k*_ = 0.0. Comparing this panel to the upper right panel in Figure 7, we see that the downward spike seen for *n* = 2 opens up, becomes wider, and moves toward more negative values of *k*_0_ for *n* = 9. A more subtle observation is that the migration of the surfaces in all the panels seems to occur slightly slower with respect to Δ_*k*_. By this we mean that for equal surface migration, the *n* = 9 case may require a slightly higher value of Δ_*k*_ than for the *n* = 2 case. Overall, however, we see qualitative agreement with our results for *n* = 2. Specifically, our observation that increasing the synaptic strength diversity causes the various surfaces to migrate toward regions of the parameter space with larger and/or non-physical values of the parameters is consistent with the *n* = 9 results shown in Figure 10.

## 4. Discussion

We constructed a large network of theta neurons that included diversity in the excitability parameters as well as connections with diversity in their coupling strengths. Our aim was to examine the effects of adding this synaptic diversity. Extending previous work in Luke et al. (2013), we applied the OA reduction technique to derive a surprisingly simple ordinary differential equation that we used to identify the asymptotic behavior of the order parameter, which quantifies the macroscopic collective behavior of the network. Setting the synaptic diversity to zero, we constructed one-dimensional bifurcation diagrams and found dynamical structures that underlie the repertoire of collective behaviors that the network exhibits: equilibrium states—both nodes and foci—corresponding to states of partial synchrony of the network, limit-cycle states of temporally-evolving partial synchrony, saddle-node, Hopf, and homoclinic bifurcations, node-focus transitions, and different versions of multistability (Luke et al., 2013). We then increased the synaptic diversity and found that these rich dynamical structures migrated away toward unphysical and/or extreme regions of parameter space, except for one portion of the node-focus transition surface.

It is interesting to note that Ott and Antonsen's analysis revealed how the potentially high-dimensional behavior of a population of phase oscillators collapses onto a low-dimensional “OA manifold” defined by their ansatz (Equation 13) (Ott and Antonsen, 2008). But this does not happen in networks of identical phase oscillators. In fact, the OA manifold is only attracting when the oscillator population is heterogeneous (Ott and Antonsen, 2009; see also Pietras and Daffertshofer, 2016, which addresses this issue for systems such as our theta neuron network). Indeed, it becomes more attracting with increasing parameter heterogeneity. Accordingly, we found that incorporating an additional dimension of diversity into our network resulted in even simpler behavior than we already had.

Our sequences of one- and three-dimensional bifurcation diagrams allow us to interpret this somewhat abstract description of the complexity collapse within the more concrete context of our specific network, and draw inferences in biophysical terms. Dynamical complexity arises from macroscopic bifurcations, which require the right mix of parameters such that different dynamical tendencies compete against each other (Luke et al., 2013). We grossly categorized these into two cases: Case 1 corresponds to predominantly resting but excitable neurons connected mostly by excitation, and Case 2 corresponds to predominantly spiking neurons connected mostly by inhibition (the qualifying adjectives are necessary because the Cauchy–Lorentz distribution has infinite support). These scenarios have been studied for decades; for a small sampling, see, e.g., (Van Vreeswijk et al., 1994; Hansel et al., 1995; Brunel and Hakim, 2008), and some recent works (Devalle et al., 2017; di Volo and Torcini, 2018; Bi et al., 2020) that have investigated mechanisms for the emergence of collective oscillations in Case 2, as we discuss below. Note that our two cases suffice: parameter space regions corresponding to other mixtures of parameters were less interesting in that they did not contain bifurcations.

With Δ_*k*_ = 0, we see from the first panels in Figures 1, 2, and 6 that saddle-node bifurcations, unstable equilibria, and multistability between different equilibria occur in both Cases 1 and 2. In addition, the region inside the V-shape of the folded saddle-node surfaces, where multistability occurs, is wider if Δ_η_ is smaller, meaning that in both cases, a narrower distribution of neuronal excitability favors multistability. Most importantly, adding diversity by increasing Δ_*k*_ causes the two saddle-node surfaces to move away from each other, deeper into their own regions. That is, the Case 1 surface moves toward negative η_0_ and positive *k*_0_, and the Case 2 surface moves toward positive η_0_ and negative *k*_0_. In both cases, they also move toward the unphysical region of negative Δ_η_. This migration is quite significant: within the parameter space shown in Figure 6 (i.e., η_0_∈[−30, 30], *k*_0_∈[−40, 40], Δ_η_∈[0, 3]), only a tiny sliver of the Case 2 saddle-node surface remains for Δ_*k*_ = 3.0. This suggests that the Case 1 surface moves away more quickly with respect to Δ_*k*_. Indeed, for Δ_*k*_ = 6.0, to see only small slivers of both surfaces requires the much larger and asymmetric parameter space region defined by η_0_∈[−200, 100], *k*_0_∈[−60, 120], and Δ_η_∈[0, 3] (not shown). All this means that with substantial synaptic diversity, complexity in the sense of finding saddle-node bifurcations requires very carefully tuned parameters at *extreme* values.

Similarly, we see in Figure 7 that Hopf bifurcations occur only in Case 2, i.e., with predominantly spiking neurons (η_0_>0) and inhibitory synapses (*k*_0_ < 0), as is generally well-known (Van Vreeswijk et al., 1994; Hansel et al., 1995; Ermentrout, 1996; Brunel and Hakim, 2008; Devalle et al., 2017). We also find that Hopf bifurcations occur preferentially for more uniform networks (Δ_η_ small). In our theta neuron network, the vast majority of these are of the super-critical variety, but sub-critical bifurcations do occur in a small region of parameter space corresponding to weakly active neurons (small η_0_) and little synaptic diversity (small Δ_*k*_). And again, we see that with increasing synaptic diversity, the Hopf surface moves away such that this bifurcation requires more removed (η_0_≫0) and narrower (Δ_η_≳0) distributions of the excitability parameter, as well as stronger inhibitory coupling (*k*_0_≪0).

Hopf bifurcations are currently of particular interest as they give rise to periodic orbits that are thought to underlie the emergence of fast gamma oscillations in inhibitory QIF networks, as has been recently investigated (Devalle et al., 2017; Bi et al., 2020). Interestingly, Bi et al. (2020) considered QIF networks with diversity in the synaptic strengths but not in the neurons' excitabilities, and found both sub- and supercritical Hopf bifurcations. In contrast, Devalle et al. (2017) considered QIF networks with diversity in the neurons' excitabilities but not in the synaptic strengths, and found only super-critical Hopf bifurcations. Recalling the equivalence between the QIF neuron and the theta neuron, it is interesting that in our theta neuron network, which includes both kinds of diversity, we find both kinds of Hopf bifurcations. However, as noted above, the sub-critical ones occur only in a small region of parameter space and with little synaptic diversity. Also interestingly, none of the works cited above report the termination of a limit cycle via homoclinic bifurcation, as we do.

But there is another important difference between the QIF models cited above and our theta neuron network that complicates the question: the synaptic connections are modeled differently. Montbrió et al. (2015) and di Volo and Torcini (2018) used delta-function pulses and included excitability but not synaptic diversity, and did not find Hopf bifurcations^1^. Bi et al. (2020) included exponentially-decaying post-synaptic currents with a non-zero time constant τ. They found both sub- and super-critical Hopf bifurcations. Devalle et al. (2017) included excitability but not synaptic diversity, and found that supercritical Hopf bifurcations only occur with τ within a finite range greater than zero, and that sub-critical Hopf bifurcations do not occur at all. In contrast to these works, we included both excitability and synaptic diversity, and we modeled our synapse by the pulse in Equation (2) with *n* = 2 (or 9). Since this is a wide pulse, we effectively have a non-zero synaptic time constant, but note that unlike (Devalle et al., 2017; Bi et al., 2020), we do not have an additional equation governing our synaptic dynamics. Thus our network is different from any of the ones considered above. We found both sub- and super-critical Hopf bifurcations, but our subcritical ones required low excitability diversity (i.e., small Δ_η_). All of this might suggest that in addition to the requirement for a non-zero synaptic time constant, diversity in excitability might favor the occurrence of supercritical Hopf bifurcations, and synaptic diversity might favor subcritical Hopf bifurcations. But this is not clear, since in our case, the subcritical variety only occurred with small amounts of synaptic diversity. Furthermore, O'Keeffe and Strogatz (2016) studied a mixed system of excitable and active oscillators analogous to our theta neurons, and compared the effects of using a broad pulse vs. a delta-function pulse for the coupling. They found *only* subcritical Hopf bifurcations for the broad pulse coupling, and only supercritical Hopf bifurcations for the delta-function coupling.

It would be interesting to examine the limit *n* → ∞, for which our pulse approaches a delta function. Given the results in Devalle et al. (2017), we might expect the Hopf bifurcation surface to disappear in this limit. Our results for the Hopf surface with *n* = 9 (a narrower pulse), shown in the middle row of Figure 10, perhaps hints at this. Compared to the *n* = 2 case, the Hopf surface for Δ_*k*_ = 0.0 has shifted toward more negative values of *k*_0_ (stronger inhibitory coupling), especially for small Δ_η_ (narrower excitability distributions). Interestingly, however, no such overall shift appears to occur for the saddle-node or node-focus surfaces. In any case, a more complete study would certainly be needed before drawing any confident conclusions.

di Volo and Torcini (2018) and Bi et al. (2020) identified another mechanism that may give rise to slow gamma oscillations, namely fluctuation-driven oscillations that circulate around a stable focus. Since this mechanism does not work with a node, this is relevant to our study of the node-focus transition. This transition is not a true bifurcation in that it does not involve changes in either the existence or stability of a solution. Nevertheless, we identified the corresponding surfaces in parameter space, and observed that, as the synaptic diversity is increased, they behave both similarly and differently as compared to surfaces of the true bifurcations discussed above. We found (Figure 9) that in the parameter space corresponding to our Case 1, there are essentially three NF surfaces that are crossed as *k*_0_ changes—a folded upper surface with two intersections and a lower surface—thus introducing complexity in the possible network behavior. However, the upper (higher *k*_0_) folded sheet migrates away toward extreme values of negative-η_0_ as synaptic diversity increases. In contrast, the increased synaptic diversity does not cause the lower NF surface, which occurs for negative *k*_0_, to migrate away. It persists. For Case 2, only this lower NF surface occurs. Furthermore, as Δ_*k*_ increases and the saddle-node and Hopf surfaces move away, we find that the central parameter space rather neatly splits into a region for which a stable focus equilibrium exists for *k*_0_ larger than a critical value (which depends increasingly weakly on η_0_ and Δ_η_), and a stable node exists for *k*_0_ more negative than this critical value. This suggests that for non-extreme, physiologically “reasonable” parameter sets and sufficiently large fluctuations, the occurrence of fluctuation-driven collective network oscillations in networks of theta neurons with significant diversity in the connection weights depends quite simply on the value of the center of the connection weight distribution.

It is important to note that in Appendix E1 of Montbrió et al. (2015), the authors considered the same issue—the effect of introducing synaptic diversity—that we examine here. There are some differences in our formulations of the problem, however. We constructed our network using theta neurons, and they used quadratic integrate-and-fire neurons. This is not a major difference because, as was noted previously, these systems are related by a change of variable. Furthermore, we both used independent Cauchy–Lorentz distributions for the excitability parameters and synaptic strengths (i.e, Equation 3). A more important difference lies in the synaptic models. Montbrió et al. (2015) used delta function pulses, whereas we use the continuous pulse of Equation (2) with *n* = 2 (or 9), which is wide with respect to the state of the pre-synaptic neuron and is always “on” (see also O'Keeffe and Strogatz, 2016, which used a similar pulse). Also different are the macroscopic variables used to describe the collective network dynamics: We used the Kuramoto order parameter, and Montbrió et al. (2015) used the more directly interpretable quantities of firing rate and mean membrane potential. But we both found that the macroscopic equations, when extended to the case with heterogeneous coupling strengths, simply involves a single additional term proportional to the width of the coupling strength distribution.

Montbrió et al. (2015) reported their results in their Figure 9, which shows a family of saddle-node bifurcation curves parameterized by Γ/Δ^1/2^ on a two-dimensional plot of their rescaled parameters $\bar{J}/\Delta^{1/2}$ vs. $\bar{\eta }/\Delta$, where $\bar{J}$ and $\bar{\eta }$ are the center values of their current and synaptic weight distributions, and Δ and Γ are their widths, respectively. The saddle-node curves identify regions of bistability, and these are seen to shift toward lower values of $\bar{\eta }/\Delta$ and higher values of $\bar{J}/\Delta^{1/2}$ as Γ/Δ^1/2^ increases. We note that their graph is restricted to what is the equivalent of our Case 1: mostly excitable neurons with mostly excitatory connections ($\bar{\eta }/\Delta <0$ and $\bar{J}/\Delta^{1/2}>0$).

We see qualitatively equivalent behavior in our formulation: a careful study of appropriate slices of the surfaces shown in the upper panels of our Figure 3 reveals that our results are consistent with those already published in Figure 9 of Montbrió et al. (2015). However, we do not rescale our parameters as they do, and this allows us to observe that the saddle-node surfaces move toward extreme values of ±η_0_ and ±*k*_0_, and into the unphysical negative-Δ_η_ region, as we increase the synaptic strength diversity Δ_*k*_. We believe that it is appropriate to assume that the parameters η_0_, *k*_0_, and Δ_η_ have a somewhat restricted range of “reasonable” values. In this sense, our main result can be taken to mean that with increasing synaptic diversity, parameter values that correspond to interesting bifurcations of macroscopic variables move toward extreme and “unreasonable” regions of parameter space, and in this sense, are not likely to be encountered under “reasonable” circumstances. This conclusion is not evident in Figure 9 of Montbrió et al. (2015).

Furthermore, we adopt a more comprehensive view of the parameter space as compared to Montbrió et al. (2015) that includes our Case 2, i.e., networks of spiking neurons (η_0_>0) coupled by inhibition (*k*_0_ < 0), as well as the rest of the parameter space. In addition, we also consider saddle-node and Hopf bifurcations as well as the node-focus transition. See Figures 6, 7, and 9, respectively. It is interesting to note that the occurrence of saddle-node bifurcations are essentially restricted to Cases 1 and 2, and Hopf bifurcations just to Case 2, whereas the “off-diagonal” regions do not contain any bifurcation structures. We also observed that for small values of Δ_*k*_, a folded surface of node-focus transitions occurs within the “reasonable” Case 1 region of parameter space, thus adding an additional measure of complexity which shifts away to “unreasonable” regions as Δ_*k*_ increases.

In a biological sense, a rich dynamical structure represents the means by which the firing patterns of neural assemblies in the brain can be dynamic and change states in response to external stimuli. Such differences in macroscopic patterns have been shown to strongly correlate with the function of different brain regions (Shinomoto et al., 2009). At the same time, our findings are consistent with an *in vitro* study of how intrinsic heterogeneity in the phase response curve (PRC) characteristics of olfactory bulb mitral cells limits correlation-induced synchronous neural oscillations (Burton et al., 2012). See also the theoretical analysis of Pazó et al. (2019), which finds that beyond a critical level of PRC heterogeneity, the incoherent state—a simple equilibrium—is always stable. These works, and our observations reported here, suggest that evolution tunes the diversity of neuronal populations to achieve an appropriate balance between dynamical complexity and simplicity, depending on function.

Several avenues for future work suggest themselves. First, the assumption of global coupling may or may not be realistic, depending on the level of description that is desired. Interestingly, however, our network formulation includes a kind of sparsely-connected network in the case *k*_0_ = 0, in which the majority of synaptic connections are very weak, regardless of the chosen spread Δ_*k*_. This observation was used explicitly in di Volo and Torcini (2018) to relate collective oscillations in a network with a Cauchy–Lorentz distribution of in-degrees to the occurrence of a collective stable focus in the analogous globally-coupled network of Montbrió et al. (2015). In our work, we find in Figures 6, 7, and 9 that the *k*_0_ = 0 plane is the very boundary between the regions of interesting and simple dynamical structures. Second, our formulation allows a study of the role of the synaptic sharpness parameter *n*, particularly with respect to the occurrence of Hopf bifurcations, as described above. Third, it would be interesting to examine in greater depth the consequences of the different synapse models used in our work and in the various QIF networks cited above. Fourth, we assumed that the probability distributions *g*_η_ and *g*_*k*_ were independent, largely for mathematical convenience. However, fast-spiking neurons are typically inhibitory, and regularly-spiking neurons are typically excitatory, suggesting that it would be interesting to analyze our network with a more complicated joint probability distribution *g*(η, *k*). Fifth, Pazó and Montbrió (2014) applied the OA technique to study pulse-coupled oscillators described by phase response curves, an approach that makes it possible to study the role of synaptic diversity in networks of Type II neurons (Pazó et al., 2019). Sixth, previous work has shown that in populations of coupled excitable systems subjected to an external periodic driving and/or noise, a resonance effect can occur for an optimal degree of oscillator diversity (Tessone et al., 2006, 2007). Thus, extending our autonomous network to include these kinds of external inputs might yield interesting insights about the interplay between this resonance effect and our observation that diversity leads to simpler dynamics. Finally, it would be interesting to allow the coupling strength between particular neurons to evolve dynamically based on activity, and to study the conditions on the synaptic plasticity rule that lead to simple or complex dynamical structures for the network's behavior.

Understanding the brain requires studying models of neuronal network dynamics with a balance between accurate biological description and analytical tractability. Real biological networks are typically studied by recording from several neurons and studying correlations (Gerstein and Kirkland, 2001). On the other hand, mathematical studies such as ours give a quantitative understanding of the dynamical and behavioral repertoire of what these networks can do, and suggest what to look for in the laboratory.

## Data Availability Statement

Codes used to generate data for this study are available upon request to the corresponding author.

## Author Contributions

EB and PS conceived the project. LL, EB, and PS carried out the mathematical analysis and numerical simulations, and constructed the figures. LL wrote the initial manuscript. EB and PS subsequently revised it.

## Conflict of Interest

The authors declare that the research was conducted in the absence of any commercial or financial relationships that could be construed as a potential conflict of interest.

## Footnotes

^1^Montbrió et al. (2015) included synaptic diversity in their Appendix E1, but did not consider inhibitory coupling, necessary for Hopf bifurcations.
